# Exploring the anti-diabetic potential of peimisine through bioinformatics analysis and *in vitro* studies

**DOI:** 10.3389/fphar.2026.1766852

**Published:** 2026-03-27

**Authors:** Xuejing Feng, Jiayuan Jin, XiangXue Ye, Jianping Wu, Bo Yang, Qiaojun He, Peihua Luo, Jiabin Lu, Xiaochun Yang

**Affiliations:** 1 Center for Drug Safety Evaluation and Research, College of Pharmaceutical Sciences, Zhejiang University, Hangzhou, China; 2 Innovation Institute for Artificial Intelligence in Medicine of Zhejiang University, College of Pharmaceutical Sciences, Zhejiang University, Hangzhou, China; 3 Institute of Pharmacology & Toxicology, College of Pharmaceutical Sciences, Zhejiang University, Hangzhou, China; 4 School of Medicine, Hangzhou City University, Hangzhou, China; 5 Nanhu Brain-computer Interface Institute, Hangzhou, China; 6 Hangzhou Institute of Innovative Medicine, College of Pharmaceutical Sciences, Zhejiang University, Hangzhou, China

**Keywords:** bioinformatics, fritillariae cirrhosae bulbus, gluconeogenesis, Hsp90, peimisine, type 2 diabetes

## Abstract

**Ethnopharmacological relevance:**

*Fritillariae Cirrhosae Bulbus* is a traditional herb with diverse activities, yet its active metabolites against type 2 diabetes (T2D) remain unclear.

**Objective:**

This study aimed to identify key bioactive metabolites from *Fritillariae Cirrhosae Bulbus* through database mining, and to evaluate the therapeutic potential of the selected metabolite peimisine against T2D through bioinformatics and experimental validation.

**Methods:**

Metabolites were retrieved from TCMSP. Following ADME screening and literature validation, six metabolites were identified, from which peimisine was selected based on AlogP. Its targets were predicted using multiple databases, followed by GO and KEGG enrichment analyses and disease association analyses. Glucose uptake and gluconeogenesis assays were conducted in HepG2 cells, and key targets were further analyzed via PPI network and molecular docking.

**Results:**

Six metabolites were identified, with peimisine selected as the most promising candidate. Bioinformatics analysis predicted 48 potential targets, with enrichment in metabolic pathways and a strong association with T2D. Experimentally, peimisine at 20 μM increased glucose uptake by up to 36.30% and reduced medium glucose by 57.65% under normal conditions; in an insulin-resistance model, it restored uptake by 42.82% and lowered glucose by 15.32%. It also significantly suppressed gluconeogenic enzymes, reducing PEPCK mRNA by 80% and G6PD by 31% relative to control. HSP90AA1 was identified as a central target, with a docking score of −7.9 kJ/mol.

**Conclusion:**

Peimisine, a metabolite of *Fritillariae Cirrhosae Bulbus*, demonstrates anti-T2D potential by enhancing glucose uptake and suppressing gluconeogenesis, likely through targeting HSP90AA1, supporting its development as a phytotherapeutic candidate for T2D.

## 1 Introduction

*Fritillariae Cirrhosae Bulbus* (known as Chuanbeimu in Chinese) derived from several *Fritillaria* species within the family Liliaceae, is one of the traditional precious Chinese medicinal materials with a long history of medicinal use. With increasing research depth on *Fritillariae Cirrhosae Bulbus*, great progress has been made in the study of its metabolites and pharmacological effects (Hao et al., 2013). The researchers have isolated and determined alkaloids, organic acids and their esters, nucleosides, sterols and their glycosides, polysaccharides, volatile oils, phytosterols and their glycosides, and trace elements, with antitussive, expectorant, antiasthmatic, sedation and analgesia, anti-inflammatory, antibacterial, anti-oxidation, antitumor and other activities (Liu et al., 2020; Luo et al., 2019; Wang et al., 2012; Yin et al., 2019). Close attention has been paid to *Fritillariae Cirrhosae Bulbus* at present due to its potential role in the treatment of a variety of diseases, such as acute lung injury, tuberculosis, Parkinson’s disease, and so on (Ghanem et al., 2021; Liang et al., 2017; Wang et al., 2016). Nevertheless, due to the complexity of Chinese medicine metabolites, most studies about *Fritillariae Cirrhosae Bulbus* only focused on the active fraction, such as water extract, alcohol extract, total alkaloids, etc. And there is a lack of in-depth pharmacological research on *Fritillariae Cirrhosae Bulbus* in clarifying the active metabolite and exploring the underlying mechanism, which may hinder the utilization and development of *Fritillariae Cirrhosae Bulbus* resources in the field of drug discovery and disease treatment.

Traditional drug development is hampered by long timelines, exorbitant costs and a high failure rate. Natural products have attracted much attention due to their diverse structures, low toxicity and multi-target characteristics. However, their clinical transformation remains constrained by unclear targets and mechanisms. With the rapid development of computer technology, an increasing number of bioinformatics methods based on high-throughput sequencing and various omics have been applied to the basic research in TCM (Gong et al., 2018; Leung et al., 2013; Liu et al., 2015), which provide a good opportunity to discover potential targets and improve our understanding about the underlying action mechanism of TCM rapidly and efficiently.

Type 2 diabetes (T2D) is a chronic disease with high prevalence worldwide, affecting 589 million adults in 2024 and causing 3.4 million deaths. Global prevalence is projected to rise by 45%–853 million by 2050 (Duncan et al., 2025). Many serious complications of T2D pose a threat to human health, such as obesity, metabolic impairment, atherosclerosis, diabetic nephropathy and cardiovascular disease. Treatment of T2D encompasses dietary modification, physical activity and antihyperglycaemic drugs (Nauck et al., 2021). However, despite all these therapeutic measures, the complications of T2D still cannot be adequately controlled. Thus, the search for novel treatment options remains the frontier of T2D research. Although a variety of pharmacological effects of *Fritillariae Cirrhosae Bulbus* have been reported, the specific active metabolites and mechanisms of action relevant to T2D remain underexplored.

In this study, we identified peimisine as an active metabolite of *Fritillariae Cirrhosae Bulbus* through multiple databases and bioinformatics analysis. A systematic investigation of its targets, functions, and pathways revealed that peimisine has the potential to treat T2D. Further evidence of its therapeutic efficacy was demonstrated by examining the impact of peimisine on glucose uptake and gluconeogenesis in HepG2 cells. Finally, protein-protein interaction (PPI) network analysis and molecular docking were employed to identify and validate the key target. This study provides a research basis for the treatment of T2D with peimisine, and offers new insights and perspectives for the development of natural products.

## 2 Materials and methods

### 2.1 Active metabolites screening of *Fritillariae Cirrhosae Bulbus*

The Traditional Chinese Medicine Systems Pharmacology (TCMSP, http://lsp.nwu.edu.cn/tcmsp.php) was used to obtain and screen potential active metabolites of *Fritillariae Cirrhosae Bulbus*, which can provide pharmacokinetics and pharmaceutical chemical properties, such as molecular weight (MW), oral bioavailability (OB), drug-likeness (DL), blood-brain barrier (BBB) and so on (Ru et al., 2014).

OB and DL are indicators of drug screening, which play a critical role in evaluating the effectiveness of compounds in systemic circulation and similarity to known drugs (Zhang et al., 2019). Additionally, Lipinski’s “rule of five” (RO5), namely, molecular weights (MW) < 500 Da, calculated logarithmic value of n-octanol/water partition coefficient (AlogP) < 5, hydrogen-bond donors (Hdon) < 5 and hydrogen-bond acceptors (Hacc) < 10, which describes the compatibility of the drug with the human body, is one of the common rules for drug molecular design and compound screening (Lipinski, 2004).

In the present study, OB ≥ 30%, DL ≥ 0.18 and RO5 were set as screening conditions to obtain candidate metabolites of *Fritillariae Cirrhosae Bulbus*. After that, one of the candidate metabolites was determined for further bioinformatics prediction and analysis.

### 2.2 Target prediction

The structure and “Canonical SMILES” of the compound determined in the previous step were found in the PubChem database (https://pubchem.ncbi.nlm.nih.gov/) to standardize compound information (Kim et al., 2016). Considering the possibility of false positives arising from frequent hitter mechanisms, we assessed the frequent hitter potential of peimisine by submitting its SMILES structure to ChemFH (https://chemfh.labmol.com.br/) (Shi et al., 2024).

Potential targets of the metabolite were obtained from TCMSP database with default parameters, Similarity ensemble approach (SEA) database based on p-values (http://sea.bkslab.org/), ChEMBL database with default parameters (https://www.ebi.ac.uk/chembl/), Swiss Target Prediction database based on Probability (http://www.swisstargetprediction.ch/) and HTDocking web application using Docking Score ≥11 (https://www.cbligand.org/HTDocking/) (Daina et al., 2019; Keiser et al., 2007; Liu et al., 2014; Zdrazil et al., 2024). All targets predicted from these sources were pooled, and only those annotated as human proteins were retained. Then, the UniProt database (http://www.UniProt.org/) was used to standardize targets information to their standard gene names and the Ensembl Gene ID for facilitating subsequent enrichment analysis and network construction (Han et al., 2021).

### 2.3 Target gene function and pathway enrichment analysis

Gene enrichment analysis can be utilized to thoroughly comprehend the effects of these putative targets in biological functions and signaling pathways. Gene Ontology (GO) functional enrichment analysis and Kyoto Encyclopedia of Genes and Genomes (KEGG) pathway analysis were performed using the OmicShare tools, a free online platform for data analysis (www.omicshare.com/tools) (Li et al., 2021).

Gene Ontology (GO) is an international standardized gene functional classification system that offers a dynamic-updated controlled vocabulary and a strictly defined concept to comprehensively describe the properties of genes and their products in any organism. GO enrichment analysis can provide all GO terms with significant enrichment of differentially expressed genes (DEGs) compared to the genome background and filter the DEGs that correspond to biological functions. Firstly the standard target genes, as DEGs, were mapped to GO terms in the Gene Ontology database, gene numbers were calculated for every term. Then, GO terms meeting corrected-*P* value ≤ 0.05 were defined as significantly enriched GO terms in DEGs (Zhao et al., 2019).

KEGG is the major public pathway-related database. Similar to GO enrichment analysis, Pathway enrichment analysis can identify significantly enriched metabolic pathways or signal transduction pathways in DEGs compared with the whole genome background (Kanehisa et al., 2017).

### 2.4 Targets to diseases prediction and network construction

The Database for Annotation, Visualization and Integrated Discovery (DAVID) (https://david.ncifcrf.gov/) is a bioinformatics and online analysis database, which provides a comprehensive set of functional annotation tools for investigators to understand the biological meaning behind a large list of genes (Dennis et al., 2003).

Potential candidate target genes were input and formed a gene list used for functional annotation analysis in DAVID v6.8. Then, the annotation summary results including disease prediction results from the Genetic Association Database (GAD) in DAVID were obtained. The diseases with the high correlation degree were selected according to the *P* value ≤ 0.1 (Huang et al., 2009).

In order to understand the complex relationships among targets and diseases, the processed data from the DAVID database were imported into Cytoscape 3.8.2 software to construct a visualized targets-diseases network (Ding et al., 2021).

### 2.5 Cell culture and treatments

Human hepatocellular carcinomas HepG2 cells were purchased from Chinese Academy of Sciences (Shanghai, China) and were cultured in DMEM (318000, Gibco, California, USA) containing 10% FBS (A5669701, Gibco, California, USA) and 100 U/ml penicillin and streptomycin at 37 °C in a humidified incubator with 5% CO2. HepG2 cells were treated with different concentrations of peimisine (S20082, purity 99.2%, Yuanye Bio-Technology, Shanghai, China) with or without palmitic acid (PA, H8780, Solarbio, Beijing, China) for 24 h, followed by the biochemical analyses described below.

### 2.6 Cell viability assay

HepG2 cells were seeded into 96-well plates and cultured overnight. Cells were treated with PA or different concentrations of peimisine, as previously described. Then 10% Cell Counting Kit-8 (CCK8) (C0005, Topscience Co. Ltd., Shanghai, China) was added to each well and incubated for 1 h at 37 °C. Finally, the absorbance was measured at 450 nm using a microplate meter (Spark, TECAN, Switzerland).

### 2.7 Glucose consumption assay

HepG2 cells were seeded into 96-well plates and treated with different concentrations of peimisine with or without PA for 24 h. Insulin solution (S12033, Yuanye Bio-Technology, Shanghai, China) was added to a final concentration of 1 μM and incubated for 0.5 h. According to the manufacturer’s instructions, the glucose concentrations in the culture medium were measured by the glucose assay kit (A154-one to one, Jiancheng Bioengineering Company, Nanjing, China) to reflect the glucose consumption by the cells.

### 2.8 Glucose uptake assay

HepG2 cells were seeded into 96-well plates and treated with different concentrations of peimisine with or without 250 μM PA for 24 h. After treatment, the cells were incubated with insulin solution at a final concentration of 1 μM for 0.5 h, then replaced with a serum-free medium containing 200 μM of 2-[N-(7-Nitrobenz-2-oxa-1,3-diazol-4-yl)amino]-2-deoxy-D-glucose (2-NBDG) (ST2078, Beyotime, Shanghai, China) and incubated for 30 min at 37 °C in the dark. Finally, the cells were washed twice with PBS, and the fluorescence intensity at Ex/Em = 485/535 nm was measured using a microplate meter (Spark, TECAN, Switzerland).

### 2.9 Quantitative real time polymerase chain reaction

Total mRNA was extracted using FastPure Cell/Tissue Total RNA Isolation Kit V2 according to the manufacturer’s instructions (RC112-01, Vazyme Biotech Co., Ltd., Nanjing, China). cDNA was prepared using a cDNA reverse transcription kit (AT311, TransGen Biotech, Beijing, China). Quantitative real-time polymerase chain reaction (qPCR) was carried in a CFX96TM Real-Time System (Bio-Rad Hercules CA USA) using TB Green Premix Ex Taq (Tli RNaseH Plus) (RR420A, Takara, Tokyo, Japan). The primer sequences were as follows:

PEPCK-forward, 5′- AGT​AGA​GAG​CAA​GAC​GGT​GAT-3’; PEPCK-reverse, 5′- TGC​TGA​ATG​GAA​GCA​CAT​ACA​T-3’; G6PD-forward, 5′- TCC​TCA​AGA​ACC​TGG​GCA​CG-3’; G6PD-reverse, 5′-CTA​CAA​TAG​AGC​TGA​GGC​GG-3’; ACTB-reverse, 5′-ACC​CTG​AAG​TAC​CCC​ATC​GAG-3’; ACTB-reverse, 5′-AGC​ACA​GCC​TGG​ATA​GCA​AC-3’.

### 2.10 Construction and analysis of protein-protein interaction network

A thorough understanding of PPI clarifies the roles of individual proteins and the complex cellular circuits that lead to disease (Nada et al., 2024). Disease-related targets enriched via the DAVID database were used to construct a PPI network in the string database (https://string-db.org/). Cytoscape 3.8.2 software was utilized to visualize the interaction network and the “NetworkAnalyzer” function in the Cytoscape 3.8.2 software was used for topology analysis.

### 2.11 Molecular docking

Crystal structures of HSP90AA1 (PDB ID: 5H22) was obtained from PDB database (https://www.rcsb.org/) (Burley et al., 2023). The 3D structure of the peimisine was downloaded from PubChem (https://pubchem.ncbi.nlm.nih.gov/) (Kim et al., 2021). AutodockTools was used to prepare the crystal structure of the target protein. The ligands were processed for energy minimization. AutoDock Vina was used to dock peimisine and HSP90AA1 to obtain binding energies (Trott and Olson, 2010). PyMOL was applied to visualize the docking status.

### 2.12 Statistical analysis

All cell experiments were performed with at least three independent biological replicates, and the data were presented as mean ± standard deviation. The normality of data and homogeneity of variances were assessed by the Shapiro–Wilk test and Levene’s test, respectively. For data complying with both assumptions, one-way analysis of variance (ANOVA) and the LSD test were used to compare the mean differences among multiple groups; otherwise, the non-parametric Kruskal–Wallis test was applied. A difference was considered statistically significant when *p* < 0.05.

## 3 Results

### 3.1 Identification of active metabolites

The 63 metabolites of *Fritillariae Cirrhosae Bulbus*, along with absorption, distribution, metabolism and excretion (ADME) data for each metabolite were obtained using the TCMSP database. These metabolites were first screened based on OB ≥ 30%, DL ≥ 0.18 and RO5 and then further assessed through literature review to prioritize those with documented relevance to *Fritillaria* species (He et al., 2022; Wang et al., 2025; Yu et al., 1992). Through this process, six metabolites were ultimately selected for subsequent analysis (Table 1).

**TABLE 1:** Pharmacological and molecular properties from TCMSP database.

Molecule name	MW	AlogP	Hdon	Hacc	OB(%)	DL	Caco-2	BBB	FASA	HL
Peimisine	**427.69**	**3.16**	**2**	**4**	**57.40**	**0.81**	**0.18**	**−0.45**	**58.56**	**14.39**
Verticinone	429.71	3.21	2	4	60.07	0.67	0.42	−0.29	60.77	7.07
Isoverticine	431.73	3.40	3	4	48.23	0.67	0.27	−0.44	63.93	7.90
Chuanbeinone	413.71	4.23	1	3	41.07	0.71	0.61	0.08	40.54	8.07
Zhebeirine	413.71	4.23	1	3	46.96	0.71	0.76	0.24	40.54	8.49
Songbeinone	413.71	4.23	1	3	45.35	0.71	0.63	0.02	40.54	7.64

Values in bold indicate the compound peimisine, which was selected as the primary candidate for further investigation in this study.

**FIGURE 1:**
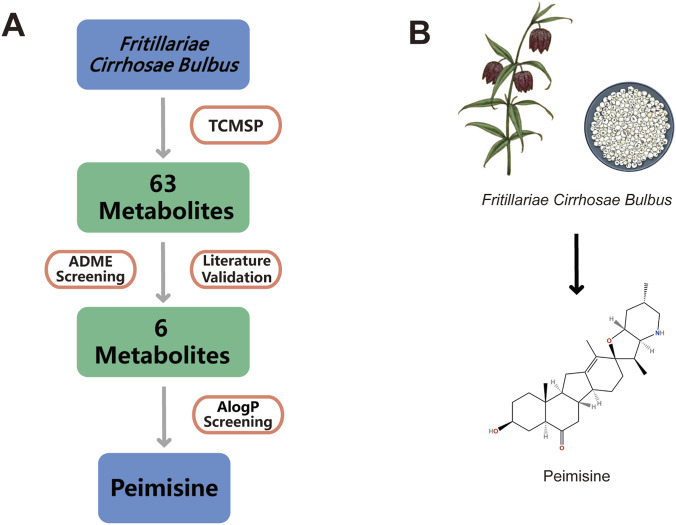
Potential activeActive metabolites constituents identification of *Fritillariae Cirrhosae Bulbus* **(A)** Workflow for the identification of potential active metabolites from *Fritillariae Cirrhosae Bulbus*. **(B)** The chemical structure of peimisine.

In the RO5, AlogP is a very important drug-like property used to evaluate the lipophilicity of candidate drugs (Pollastri, 2010). Drugs possess certain lipophilicity to combine with cell membrane or protein to function, but if the lipophilicity is too high, drug molecules will combine with multiple targets, resulting in increased broad host property, enhanced toxicity, and poor solubility and metabolic clearance (Cronin, 2006; van de Waterbeemd et al., 2001). It was reported that the average calculated logP value of the marketed oral drugs from 1985 to 2002 was about 2.5, after which the average value of small molecule drugs increased but not significantly, with a trend that metabolites with relatively low AlogP values are prone to enter the next stage of drug development (Wenlock et al., 2003). Hence, peimisine with the smallest AlogP value that is greater than 2.5 became the focus of attention among the six metabolites. Moreover, peimisine is an important alkaloid metabolite with a variety of pharmacological activities according to the existing pharmaceutical researches (Wang et al., 2012). Consequently, peimisine with great potential and value for drug discovery was selected to conduct further bioinformatics investigation and analysis. The chemical structure of peimisine is shown in Figure 1B.

### 3.2 Target prediction and enrichment analysis

#### 3.2.1 Targets prediction of peimisine

To exclude false-positive artifacts, the chemical structure of peimisine was evaluated using ChemFH. The analysis showed that peimisine passed frequent hitter rules, including PAINS, ALARM NMR, BMS and so on. In addition, no potential interference was detected across frequent hitter mechanisms (Table 2). These results indicate that peimisine is unlikely to act as a frequent hitter, thereby supporting the reliability of its subsequent target prediction and pharmacological evaluation. Potential targets of peimisine were predicted using five databases as described in the “Methods” Section 2 related human genes were identified by the TCMSP database. Three predicted target genes were obtained by removing the non-human genes in the SEA Search Server database. The target genes related to peimisine with “probability value” in the top three were selected from the Swiss Target Prediction database. 30 predicted target genes with Docking Score≥11 were selected after removing the non-human genes in the HTDocking. 13 potential targets were selected in the ChEMBL database, according to the following conditions: confidence 90% value is ‘active’ and the organism is *Homo sapiens*. To sum up, 48 assumed targets were obtained after removing duplicates (Table 3).

**TABLE 2:** ChemFH prediction of frequent hitter mechanisms for peimisine

Mechanism	Pred. score	Decision	Uncertainty	Num. of substructure	Substructure
Colloidal aggregators	0.016	Pass	High-confidence	0	-
FLuc inhibitors	0.000	Pass	High-confidence	0	-
Blue fluorescence	0.004	Pass	High-confidence	0	-
Green fluorescence	0.000	Pass	High-confidence	0	-
Reactive compounds	0.180	Pass	Low-confidence	0	-
Promiscuous compounds	0.014	Pass	High-confidence	0	-
Other assay interference	0.000	Pass	High-confidence	0	-

**TABLE 3:** Potential targets of peimisine

Target gene	Protein name	Ensembl gene ID
NR3C2	Mineralocorticoid receptor	ENSG00000151623
NR3C1	Glucocorticoid receptor	ENSG00000113580
EBP	3-Beta-hydroxysteroid-Delta (8), Delta (7)-isomerase	ENSG00000147155
GLI1	Zinc finger protein GLI1	ENSG00000111087
SHH	Sonic hedgehog protein	ENSG00000164690
HSD17B3	Estradiol 17-beta-dehydrogenase 3	ENSG00000130948
CHRNB4	Neuronal acetylcholine receptor subunit beta-4	ENSG00000117971
BRD4	Bromodomain-containing protein 4	ENSG00000141867
HSP90AA1	Heat shock protein HSP 90-alpha	ENSG00000080824
KDM1A	Lysine-specific histone demethylase 1A	ENSG00000004487
PTGER2	Prostaglandin E2 receptor EP2 subtype	ENSG00000125384
GABRA1	Gamma-aminobutyric acid receptor subunit alpha-1	ENSG00000022355
APH1A	Gamma-secretase subunit APH-1A	ENSG00000117362
ROS1	Proto-oncogene tyrosine-protein kinase ROS	ENSG00000047936
NAMPT	Nicotinamide phosphoribosyltransferase	ENSG00000105835
SPHK2	Sphingosine kinase 2	ENSG00000063176
PIN1	Peptidyl-prolyl cis-trans isomerase NIMA-interacting 1	ENSG00000127445
SMO	Smoothened homolog	ENSG00000128602
BST1	ADP-ribosyl cyclase/cyclic ADP-ribose hydrolase 2	ENSG00000109743
DYRK2	Dual specificity tyrosine-phosphorylation-regulated kinase 2	ENSG00000127334
AKR1C3	Aldo-keto reductase family 1 member C3	ENSG00000196139
CERT1	Ceramide transfer protein	ENSG00000113163
FKBP1A	Peptidyl-prolyl cis-trans isomerase FKBP1A	ENSG00000088832
CYP51A1	Lanosterol 14-alpha demethylase	ENSG00000001630
CALCRL	Calcitonin gene-related peptide type 1 receptor	ENSG00000064989
RORA	Nuclear receptor ROR-alpha	ENSG00000069667
PTGR2	Prostaglandin reductase 2	ENSG00000140043
CFTR	Cystic fibrosis transmembrane conductance regulator	ENSG00000001626
NPC1	NPC intracellular cholesterol transporter 1	ENSG00000141458
ADHX	Alcohol dehydrogenase class-3	ENSG00000197894
CYP2C9	Cytochrome P450 2C9	ENSG00000138109
PDE3B	cGMP-inhibited 3′,5′-cyclic phosphodiesterase B	ENSG00000152270
PRKCQ	Protein kinase C theta type	ENSG00000065675
ALOX5AP	Arachidonate 5-lipoxygenase-activating protein	ENSG00000132965
LTA4H	Leukotriene A-4 hydrolase	ENSG00000111144
HSD11B1	Corticosteroid 11-beta-dehydrogenase isozyme 1	ENSG00000117594
CETP	Cholesteryl ester transfer protein	ENSG00000087237
PAH	Phenylalanine-4-hydroxylase	ENSG00000171759
PNLIP	Pancreatic triacylglycerol lipase	ENSG00000175535
EGFR	Epidermal growth factor receptor	ENSG00000146648
DYRK1A	Dual specificity tyrosine-phosphorylation-regulated kinase 1A	ENSG00000157540
PRKCA	Protein kinase C alpha type	ENSG00000154229
CD1A	T-cell surface glycoprotein CD1a	ENSG00000158477
CRAT	Carnitine O-acetyltransferase	ENSG00000095321
SAT1	Diamine acetyltransferase 1	ENSG00000130066
CD1D	Antigen-presenting glycoprotein CD1d	ENSG00000158473
GBA	Lysosomal acid glucosylceramidase	ENSG00000177628
NOS3	Nitric oxide synthase, endothelial	ENSG00000164867

#### 3.2.2 Gene function and pathway enrichment analysis

**FIGURE 2:**
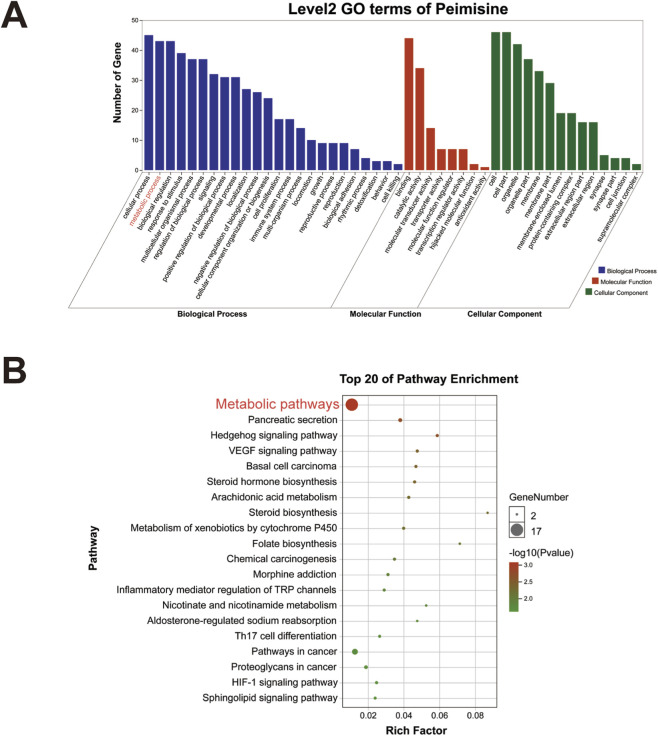
Gene function and pathway enrichment analysis **(A)** GO enrichment analysis. GO terms: Biological Processes (Blue), Molecular Function (Red) and Cellular Component (Green). **(B)** KEGG enrichment analysis. The color of the dot indicates the size of its *P* value, and the size of the dot indicates the number of genes enriched in the pathway.

GO analysis includes the three parts: biological process (BP), molecular function (MF) and cellular component (CC), and each part has its secondary GO categories. Figure 2A reveals that the 48 targets were mainly associated with cell, cell part, cellular process, binding, metabolic process, biological regulation, organelle, response to stimulus, multicellular organismal process and regulation of biological process. To further explore the potential signaling pathways of peimisine, KEGG enrichment analysis was performed. As shown in Figure 2B, the gene number and p-value indicate that peimisine is primarily enriched in metabolic pathways.

#### 3.2.3 Targets to diseases prediction of peimisine and network analysis

**FIGURE 3:**
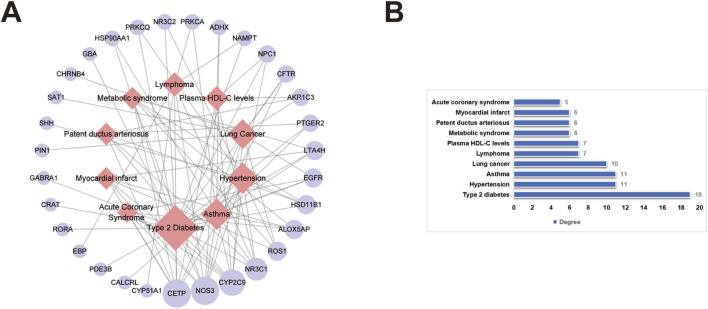
Targets-diseases network analysis of peimisine **(A)** Targets-diseases interaction network of peimisine. The size of the node indicates the size of the “degree” value, and the light red diamonds and lavender circles correspond to diseases and target genes, respectively. **(B)** Targets-diseases network topological parameter. The x-axis shows the “degree” value of the predicted diseases, and the y-axis represents the classifications of predicted diseases.

The 48 target genes were enriched and analyzed in the DAVID database to explore the possibility of disease treatment. The top 10 related diseases and their corresponding target genes were shown in Table 4, including asthma, myocardial infarct, patent ductus arteriosus, hypertension, acute coronary syndrome, lung cancer, T2D, plasma high density lipoprotein cholesterol (HDL-C) levels, lymphoma and metabolic syndrome. And then, the Cytoscape software was used to map the targets-diseases network to visualize the results (Figure 3A). Each node in the network shows a different size according to its “degree” value that is a key topological parameter obtained after network analysis (Figure 3B) (Zhao and Liu, 2019). The results of network analysis showed that the node of T2D emerged as the largest with the highest degree, indicating that peimisine possesses strong potential for treating T2D.

**TABLE 4:** Targets to diseases prediction from DAVID database.

Disease	*P* value	Genes
Asthma	2.40E-06	ALOX5AP, EGFR, PTGER2, NR3C1, CFTR, ADHX NOS3, PRKCA, CYP2C9, LTA4H, HSP90AA1
Myocardial infarct	5.28E-05	ALOX5AP, CETP, NOS3, ROS1, CYP2C9, LTA4H
Patent ductus arteriosus	7.43E-05	PTGER2, HSD11B1, NR3C1, CETP, CYP2C9, AKR1C3
Hypertension	9.72E-05	HSD11B1, NR3C1, SHH, CETP, NR3C2, PRKCQ, NOS3, ROS1, NPC1, CYP2C9, CALCRL
Acute coronary syndrome	1.17E-04	ALOX5AP, EGFR, CETP, NOS3, CYP2C9
Lung cancer	1.38E-04	CHRNB4, EGFR, CFTR, CETP, NOS3, ROS1, CYP2C9 AKR1C3, SAT1, PIN1
Type 2 diabetes	1.46E-04	GABRA1, PTGER2, CRAT, RORA, PDE3B, CFTR, NAMPT, NR3C2, PRKCQ, GBA, CYP2C9, LTA4H HSP90AA1, EGFR, HSD11B1, NR3C1, CETP, NOS3, NPC1
Plasma HDL cholesterol levels	1.80E-04	NR3C1, ADHX, CETP, NOS3, NPC1, CYP2C9, AKR1C3
Lymphoma	1.80E-04	NR3C1, NAMPT, CETP, NOS3, EBP, PRKCA, CYP51A1
Metabolic syndrome	2.28E-04	ALOX5AP, HSD11B1, CETP, NOS3, ROS1, LTA4H

### 3.3 Glucose uptake and gluconeogenesis

#### 3.3.1 Effects of peimisine on glucose uptake of HepG2

**FIGURE 4:**
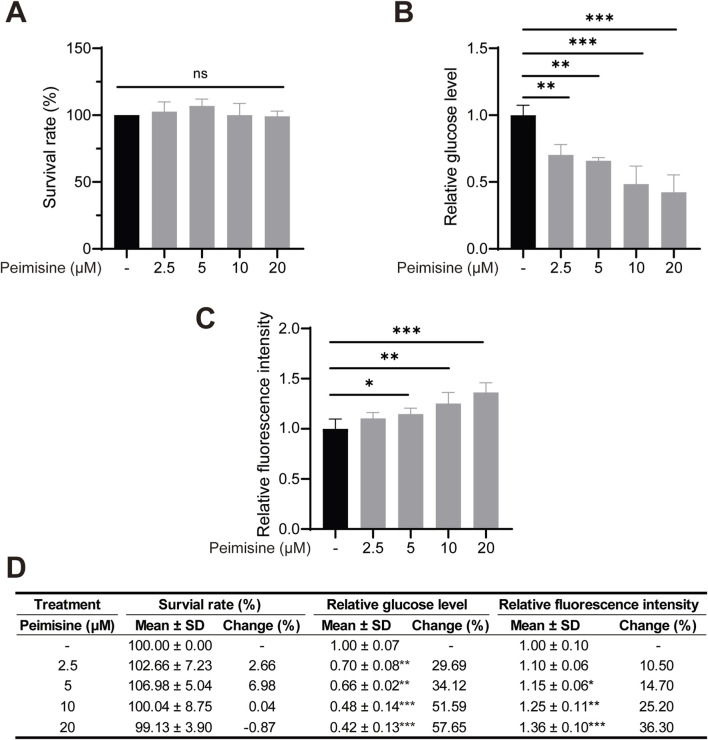
The effects of peimisine on glucose uptake in HepG2 cells **(A)** Cell viability with peimisine treatment for 24 h. **(B)** Relative glucose levels in the culture medium after peimisine treatment of HepG2 cells were normalized with the control group. **(C)** Relative fluorescence intensity of HepG2 cells treated with peimisine. **(D)** Detailed quantitative data of A-C, including cell survival rate, relative glucose level in culture medium, and relative 2-NBDG fluorescence intensity in HepG2 cells treated with different concentrations of peimisine. Data are shown as mean ± SD and percentage change relative to the control group (n = 3 per group). Multiple group comparisons were performed by one-way ANOVA followed by the LSD test. *p < 0.05, **p < 0.01, ***p < 0.001; ns, no significance.

HepG2 cells were treated with different concentrations of peimisine for 24 h, and the cell viability was detected using CCK8 to exclude the cytotoxic effect of peimisine on liver cells. The results showed that peimisine at or below 20 µM exerted no cytotoxicity to HepG2 cells (Figure 4A). For glucose-consumption measurements, HepG2 cells were treated with different concentrations of peimisine for 24 h, and the glucose content in the culture medium was detected after insulin stimulation. As shown in Figures 4B,D, peimisine treatment significantly reduced extracellular glucose content in a dose-dependent manner. The relative glucose level decreased from 1.00 ± 0.07 in the control group to 0.42 ± 0.13 with 20 μM peimisine, representing decreases of more than 50%, indicating a pronounced enhancement of glucose consumption. 2-NBDG is a fluorescently labeled deoxyglucose analogue used to directly monitor glucose uptake in living cells (Shamshoum et al., 2023). To directly visualize uptake, HepG2 cells were further incubated with 2-NBDG following insulin stimulation. The fluorescence assay demonstrated a dose-dependent increase in glucose uptake in HepG2 cells treated with peimisine. The relative fluorescence intensity rose from 1.00 ± 0.10 in the control group to 1.36 ± 0.10 at 20 μM peimisine, representing an increase of approximately 36.30% at the highest concentration tested. These results confirm that peimisine effectively enhances glucose uptake without impairing cell viability (Figures 4C,D).

#### 3.3.2 Effects of peimisine on glucose uptake of IR-HepG2

**FIGURE 5:**
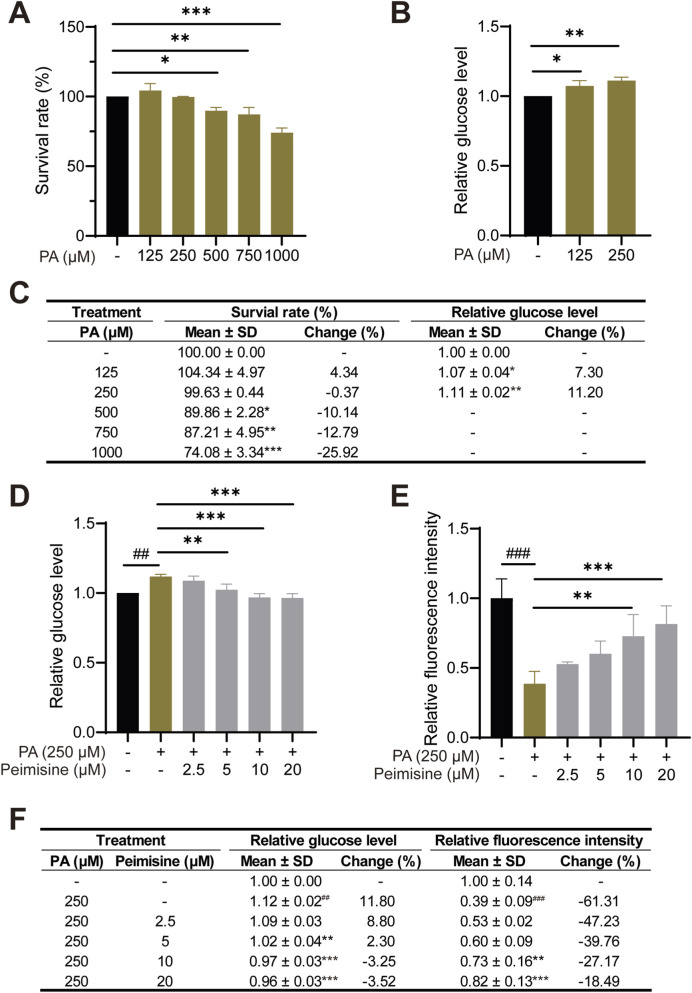
The effects of peimisine on glucose uptake in IR-HepG2 cells **(A)** Cell viability with PA treatment for 24 h. **(B)** Relative glucose levels in the culture medium after PA treatment were normalized with the control group. **(C)** Detailed quantitative data of A-B, including cell survival rate and relative glucose level in culture medium after PA treatment. **(D)** Relative glucose levels in the culture medium after peimisine treatment of IR-HepG2 cells were normalized with the control group. **(E)** Relative fluorescence intensity of IR-HepG2 cells treated with peimisine. **(F)** Detailed quantitative data of C-D, including relative glucose level and relative 2-NBDG fluorescence intensity in IR-HepG2 cells treated with peimisine. Data are shown as mean ± SD and percentage change relative to the control group (n = 3 per group). Multiple group comparisons were performed by one-way ANOVA followed by the LSD test. ##*p* < 0.001, ###*p* < 0.001 (vs. Control group); *p < 0.05, **p < 0.01, ***p < 0.001 (vs. PA group).

PA was used to induce insulin resistance (IR) in HepG2 cells. To determine the optimal concentration of PA for inducing IR, HepG2 cells were exposed to increasing concentrations of PA for 24 h. The CCK8 assays revealed that 500 μM and higher concentrations markedly reduced cell viability, whereas 250 μM PA had no cytotoxic effect yet clearly impaired insulin-stimulated glucose uptake, confirming successful induction of IR (Figures 5A–C). Therefore, 250 μM PA was selected for 24 h of treatment for the subsequent experiments.

To determine the potential effects of peimisine on glucose metabolism in PA-induced IR, glucose consumption and 2-NBDG uptake were also measured in IR-HepG2 cells. Compared with the control group, PA treatment significantly increased extracellular glucose levels by approximately 11.8% and decreased 2-NBDG fluorescence intensity by 61.31%, suggesting impaired glucose utilization. In contrast, peimisine improved glucose consumption in IR-HepG2 cells and promoted glucose uptake in a concentration-dependent manner. Following treatment with 20 μM peimisine, the relative glucose level in the culture medium decreased from 1.12 ± 0.02 to 0.96 ± 0.03, returning to a level comparable to that of the control. Concurrently, 2-NBDG fluorescence intensity significantly recovered from 0.39 ± 0.09 to 0.82 ± 0.13, representing an increase of 42.82% (Figures 5D–F). These findings demonstrate that peimisine significantly improves glucose metabolism in PA-induced IR-HepG2 cells.

#### 3.3.3 Effects of peimisine on gluconeogenesis

**FIGURE 6:**
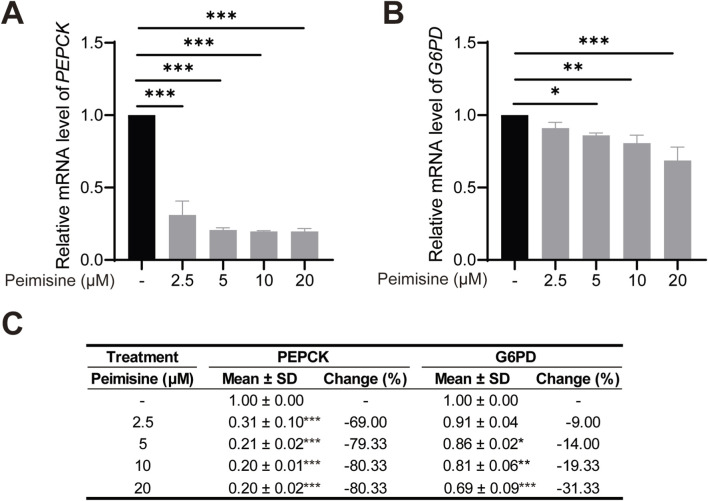
The effects of peimisine on gluconeogenesis in HepG2 cells **(A)** Relative mRNA levels of PEPCK normalized with ACTB in HepG2 cells. **(B)** Relative mRNA levels of G6PD normalized with ACTB in HepG2 cells. **(C)** Detailed quantitative data of A-B, including relative mRNA levels of PEPCK and G6PD (normalized with ACTB) in HepG2 cells treated with different concentrations of peimisine. Data are shown as mean ± SD and percentage change relative to the control group (n = 3 per group). Multiple group comparisons were performed by one-way ANOVA followed by the LSD test. *p < 0.05, **p < 0.01, ***p < 0.001.

The excessive increase of hepatic gluconeogenesis is an important cause of hyperglycemia and T2D (Xia et al., 2021). PEPCK catalyzes the conversion of oxaloacetate to phosphoenolpyruvate, and G6PD catalyzes the hydrolysis of glucose 6-phosphate to glucose. These two steps are the key rate-limiting steps in gluconeogenesis (Zhao et al., 2024). We therefore examined whether peimisine modulates these key targets in HepG2 cells. Treatment with 20 μM peimisine significantly reduced PEPCK mRNA levels by approximately 80% and suppressed G6PD expression by approximately 31%, relative to the control group. These results indicate that peimisine inhibits hepatic gluconeogenesis through attenuating expression of these rate-limiting enzymes (Figure 6).

### 3.4 PPI network and molecular docking

**FIGURE 7:**
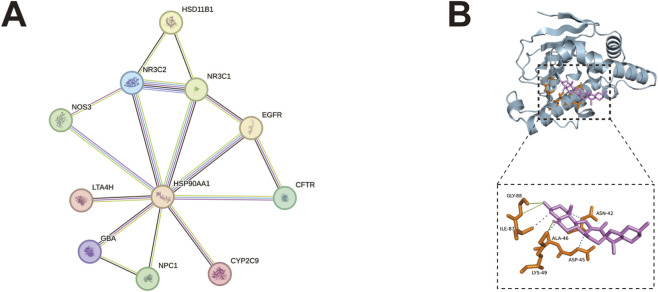
Identification of the key target with PPI network analysis and molecular docking **(A)** PPI network of intersecting targets enriched in T2D. **(B)** Molecular docking diagram of peimisine to HSP90AA1. Blue: HSP90AA1; purple: peimisine; orange: amino-acid residues interacting with peimisine; green solid lines: hydrogen bonds; blue dashed lines: hydrophobic interactions.

To explore the possible mechanism of peimisine in the treatment of T2D, the PPI network was constructed from the 19 targets that peimisine was predicted to enrich in T2D, and the disconnected genes were removed. The greater the degree value in the PPI network, the more significant the role of the protein in the network. As shown in Figure 7A, HSP90AA1 is centrally located in the PPI network. Network topology analysis revealed that HSP90AA1 exhibits superior centrality parameters, including degree, betweenness and closeness centrality, indicating that HSP90AA1 may serve as a key target for peimisine treatment (Table 5). Subsequently, we performed molecular docking analysis of peimisine with the protein encoded by HSP90AA1 with a binding energy of −7.9 kJ/mol. Typically, binding energy below −5 kJ/mol indicates robust binding between ligand and receptor (Liu et al., 2024). Peimisine forms one hydrogen bond with Ala46, interacts with Gly88 via two hydrogen bonds, and forms four hydrophobic interactions with Ile87, Lys49, Asp45, and Asn42, indicating that peimisine binds well to HSP90AA1 (Figure 7B). Based on the above, we predicted that HSP90AA1 may play a key role in the treatment of T2D with peimisine.

**TABLE 5:** Key network topology parameters of the identified hub genes in the PPI network.

Gene name	Degree	BetweennessCentrality	ClosenessCentrality
HSP90AA1	**9**	**0.759259**	**0.909091**
NR3C1	4	0.103704	0.625
NR3C2	4	0.096296	0.625
EGFR	3	0.018519	0.588235

Values in bold indicate HSP90AA1, which was identified as the central target in the protein-protein interaction network analysis.

## 4 Discussion

Peimisine is a kind of isosteroidal alkaloid, which exists in many types of *Fritillaria*, and its content in *Fritillariae Cirrhosae Bulbus* is higher than zhebeirine, chuanbeinone and so on, indicating that it may be one of the main active metabolites in the alkaloid part of *Fritillariae Cirrhosae Bulbus* (Ruan et al., 2016; Zhang et al., 2011; Zhou et al., 2008). According to the data provided by the TCMSP database in this study, all the pharmacokinetic properties of peimisine were in line with the screening conditions. And the AlogP value of peimisine was lower than that of the other five metabolites, on the basis of a higher than the average logP value of oral drugs, indicating that peimisine may have an appropriate affinity with the receptor, while it is superior to the other five metabolites in terms of selectivity, toxicity and metabolic clearance. In a word, peimisine is a valuable metabolite in *Fritillariae Cirrhosae Bulbus*, as well as a prospective candidate for drug discovery.

To evaluate the potential for non-specific interference, peimisine was systematically assessed using ChemFH, an online tool that employs deep learning models trained on large datasets of known interference compounds to identify frequent false positives and assay errors (Shi et al., 2024). The analysis demonstrated that peimisine passed the evaluated frequent hitter rules, including PAINS, ALARM NMR, and BMS, and exhibited no detectable risk across frequent hitter mechanisms such as colloidal aggregation, fluorescence, or promiscuous activity. These findings provide robust evidence that peimisine is unlikely to act as a frequent hitter, thereby supporting the specificity and credibility of its predicted target interactions and subsequent pharmacological evaluation. Subsequently, an integrated computational strategy was employed to predict potential bioactive targets of peimisine. Target prediction was performed using five databases, each applying predefined filtering criteria appropriate to its respective methodology. The results from all sources were pooled to generate a comprehensive candidate list, ultimately yielding 48 putative targets. While widely used, these computational tools have inherent limitations. Factors such as reliance on chemical similarity principles, potential biases in training data, and sensitivity to structural variations in docking algorithms can affect prediction accuracy (Hu et al., 2019). Moreover, the infrequent updating of some databases further constrains their ability to identify novel targets or mechanisms. Therefore, computational predictions should be interpreted with caution and require experimental validation.

To explore the function of peimisine, gene function and pathway enrichment analysis were performed. GO analysis results indicate that the target genes of peimisine mainly focused on metabolic process, biological regulation, stimulus response, and multicellular biological process. KEGG enrichment analysis results show that the top 20 pathways are mainly metabolism-related pathways and cancer-related pathways. Further disease prediction analysis revealed a strong association between peimisine and T2D, underscoring the therapeutic potential of peimisine for this metabolic disorder. Notably, peimisine has previously been reported to treat hypertension, asthma and lung cancer, which to some extent validates the accuracy and reliability of our disease prediction in this research (Oh et al., 2003; Wang et al., 2015; Wu et al., 2006).

As the hub of energy metabolism, the liver regulates glucose production and utilization. In T2D, the body has decreased insulin sensitivity and abnormal glucose metabolism in the liver, which inhibits glucose uptake and glycogen synthesis and promotes gluconeogenesis, giving rise to insulin resistance (Zhao et al., 2024). Gluconeogenesis refers to the conversion of non-sugar substrates to glucose and is regulated by key enzymes such as PEPCK and G6PD. Lowering blood glucose can be achieved clinically by inhibiting hepatic gluconeogenesis in T2D patients (Madiraju et al., 2014). Our findings demonstrated that peimisine dose-dependently enhanced glucose consumption and uptake, and improved insulin sensitivity in both normal and PA-induced IR-HepG2 cells. Peimisine significantly downregulated the transcription levels of PEPCK and G6PD in HepG2 cells, thereby suppressing hepatic gluconeogenesis. Collectively, these results indicated that peimisine exerts therapeutic effects on T2D by promoting glucose uptake and inhibiting gluconeogenesis in the liver.

Heat shock protein 90 (HSP90) is an ATP dependent molecular chaperone that ensures the correct folding of hundreds of client protein substrates, playing an important role in multiple biological processes. Studies have shown that the level of HSP90 in diabetes patients is elevated, and the expression of HSP90AA1 is significantly increased in the rat model of metabolic syndrome induced by high-fat diet-streptozotocin (HFD-STZ) (Hoter et al., 2018; Mishra et al., 2025). HSP90 is essential for the folding, ligand binding, and nuclear translocation of glucocorticoid receptors. Corticosteroids can regulate hepatic gluconeogenesis by modulating the transcription levels of PEPCK and G6PD (Patel et al., 2014). It has been reported that HSP90 inhibitor reversed hyperglycemia in db/db diabetes mice and improved insulin sensitivity in insulin resistant mice induced by high-fat diet (Lee et al., 2013). In order to clarify the possible mechanism of peimisine in the treatment of T2D, we constructed the PPI network from the 19 targets enriched in T2D. “Degree”, the important topological parameter, indicates the number of neighboring nodes per node in the network, and the node with a high “degree” value is the central node of the whole network (Zhao and Liu, 2019). HSP90AA1 exhibited the highest degree and was identified as the most core node, suggesting it as a key target. Molecular docking revealed that peimisine has strong affinity for HSP90AA1 and can interact with multiple amino acid residues to form hydrogen bonds, which are a relatively strong interaction force. These results suggest that peimisine may exert inhibitory effects by binding to HSP90AA1 to achieve the treatment of T2D.

An increasing number of natural compounds have recently been characterized for their potential in anti-T2D. Berberine, an isoquinoline alkaloid derived from *Coptis chinensis*, exerts anti-diabetic effects by modulating glucose and lipid metabolism, improving insulin sensitivity, and enhancing energy expenditure (Kong et al., 2020). However, its therapeutic application is constrained by low oral bioavailability (Habtemariam, 2020). Similarly, flavonoids such as quercetin exhibit notable anti-diabetic and anti-inflammatory activities, yet their poor aqueous solubility, low bioavailability, and short half-life limit their systemic utility (Miao et al., 2023). In contrast, peimisine demonstrates favorable pharmacological properties, including moderate lipophilicity in accordance with Lipinski’s rule of five. These characteristics suggest that peimisine may serve as a promising isosteroidal alkaloid scaffold for further development in T2D drug discovery.

While this study provides mechanistic insights, several limitations must be considered. First, the findings are currently derived exclusively from *in vitro* models, which cannot fully capture the systemic complexity of T2D *in vivo*. Therefore, the therapeutic potential of peimisine requires rigorous validation in established preclinical models, such as HFD/STZ-induced or db/db mice, to confirm *in vivo* efficacy, safety, and the actual pharmacokinetic profile. Second, although modern bioinformatics methods and molecular docking support HSP90AA1 as a potential direct target, further biochemical experiments are still needed to elucidate how peimisine specifically interacts with HSP90AA1 and how this interaction alleviates diabetes. Third, the inherent limitations of network analysis must be acknowledged. As recently highlighted, current database-driven approaches are prone to “output homogeneity,” where disparate studies converge on a narrow set of metabolites, targets, and pathways due to input-level biases (database incompleteness, PAINS contamination) and model-level biases (over-reliance on degree centrality, pathway enrichment size preferences) (Diao et al., 2026). In the present study, efforts were made to mitigate these concerns—including literature validation, ChemFH assessment to evaluate frequent hitter potential, multi-parameter network topology analysis, and experimental validation of core findings—which may enhance the reliability of the computational predictions. Nonetheless, computational predictions remain inherently exploratory and should be interpreted with caution.

Taken together, advancing peimisine beyond the present findings would entail addressing these specific research gaps as part of the broader pathway for translating a phytochemical into a drug candidate, which further includes challenges such as optimizing bioavailability and ensuring pharmaceutical-grade reproducibility (Popovic and Rijkers, 2025). Nevertheless, the demonstrated bioactivity against key T2D pathways, along with its promising pharmacokinetic properties, positions peimisine as a promising candidate worthy of further systematic investigation.

## 5 Conclusion

**FIGURE 8:**
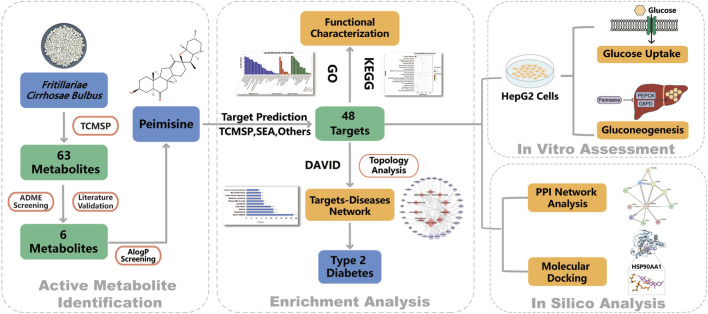
Workflow of identifying peimisine from *Fritillariae Cirrhosae Bulbus* as an anti-diabetic intervention through efficacy and mechanistic study. Candidate metabolites were screened from *Fritillariae Cirrhosae Bulbus* to identify peimisine as the key metabolite. Potential targets were predicted and subjected to enrichment and disease association analyses. *In vitro* validation in HepG2 cells, together with PPI network and molecular docking, was performed to elucidate the anti-diabetic mechanism of peimisine.

In summary, we have identified peimisine from *Fritillariae Cirrhosae Bulbus* as an agent that enhances glucose uptake and inhibits gluconeogenesis, potentially via HSP90AA1 (Figure 8). These findings position peimisine as a novel phytopharmaceutical candidate and provide a foundation for its future clinical development.

## Data Availability

The original contributions presented in the study are included in the article/supplementary material, further inquiries can be directed to the corresponding authors.
